# Robust Motor Imagery–Brain–Computer Interface Classification in Signal Degradation: A Multi-Window Ensemble Approach

**DOI:** 10.3390/biomimetics10120832

**Published:** 2025-12-12

**Authors:** Dong-Geun Lee, Seung-Bo Lee

**Affiliations:** Department of Medical Informatics, Keimyung University School of Medicine, 1095, Dalgubeol-daero, Dalseo-gu, Daegu 42601, Republic of Korea; qqwwas1234@gmail.com

**Keywords:** brain–computer interface (BCI), electroencephalography (EEG), signal degradation, common spatial pattern (CSP), time segmentation

## Abstract

Electroencephalography (EEG)-based brain–computer interface (BCI) mimics the brain’s intrinsic information-processing mechanisms by translating neural oscillations into actionable commands. In motor imagery (MI) BCI, imagined movements evoke characteristic patterns over the sensorimotor cortex, forming a biomimetic channel through which internal motor intentions are decoded. However, this biomimetic interaction is highly vulnerable to signal degradation, particularly in mobile or low-resource environments where low sampling frequencies obscure these MI-related oscillations. To address this limitation, we propose a robust MI classification framework that integrates spatial, spectral, and temporal dynamics through a filter bank common spatial pattern with time segmentation (FBCSP-TS). This framework classifies motor imagery tasks into four classes (left hand, right hand, foot, and tongue), segments EEG signals into overlapping time domains, and extracts frequency-specific spatial features across multiple subbands. Segment-level predictions are combined via soft voting, reflecting the brain’s distributed integration of information and enhancing resilience to transient noise and localized artifacts. Experiments performed on BCI Competition IV datasets 2a (250 Hz) and 1 (100 Hz) demonstrate that FBCSP-TS outperforms CSP and FBCSP. A paired t-test confirms that accuracy at 110 Hz is not significantly different from that at 250 Hz (*p* < 0.05), supporting the robustness of the proposed framework. Optimal temporal parameters (window length = 3.5 s, moving length = 0.5 s) further stabilize transient-signal capture and improve SNR. External validation yielded a mean accuracy of 0.809 ± 0.092 and Cohen’s kappa of 0.619 ± 0.184, confirming strong generalizability. By preserving MI-relevant neural patterns under degraded conditions, this framework advances practical, biomimetic BCI suitable for wearable and real-world deployment.

## 1. Introduction

A brain–computer interface (BCI) is a communication system that enables direct interaction between the human brain and external devices without the need for physical movement [1]. Brain activity can be monitored through various noninvasive techniques such as electroencephalography (EEG), magnetic resonance imaging (MRI), and functional MRI (fMRI) [2]. Among these, EEG-based BCI systems are particularly favored because of their convenience, user-friendliness, cost-effectiveness, and portability [3]. EEG-based BCIs have been widely applied to tasks involving sensorimotor rhythms [4], steady-state visual evoked potentials (SSVEPs) [5], and motor imagery (MI) [6], owing to their real-time processing capability. These systems are considered to hold great potential for both clinical [7] and commercial applications [8]. In particular, MI-BCIs prove particularly valuable for individuals with severe motor disabilities, supporting applications in neurorehabilitation, assistive device control, and communication systems [9].

The development of BCI technology is fundamentally rooted in biomimetics—the discipline of emulating biological systems to solve complex engineering challenges. The human brain represents one of nature’s most sophisticated information processing systems, capable of generating motor commands, processing sensory feedback, and adapting to dynamic environments with remarkable efficiency and robustness [X]. Motor imagery, in particular, reflects the brain’s ability to simulate motor actions internally without overt movement, activating similar neural substrates as actual execution [Y]. By decoding these endogenous cortical signals, MI-based BCIs effectively translate neural intentions into machine-readable commands, embodying a bio-inspired paradigm of direct brain-to-device communication. This neuro-mimetic interaction bypasses traditional motor pathways, offering transformative potential for individuals with motor impairments and establishing a new frontier in assistive biomimetic technologies [9].

BCI systems have shown considerable promise in laboratory settings with high-end equipment, dense electrode arrays, and high sampling frequencies [10]. A combined projection approach is proposed to enhance single-trial MI recognition for remote vehicle control [11], and a method is proposed to enhance multi-resolution spectral feature extraction for MI-based exoskeleton interfaces by integrating discrete wavelet packet transform and detrended variation analysis [12]. Additionally, Riemannian-geometry classifiers and lightweight deep learning architectures have demonstrated strong robustness to noise, channel variability, and other real-world nonstationarities [13]. These approaches often outperform classical pipelines under ideal laboratory conditions and represent powerful alternatives for practical BCI applications.

However, ideal conditions are rarely replicable in real-world settings [14]. Real-world EEG signals are often acquired in constrained environments or conditions, such as mobile or wearable devices, homes, and nonhospital environments, where significant signal degradation occurs because of limitations in the number of electrodes, signal stability, and computational resources [15]. The degradation is not limited to an increase in noise but also includes distributional shifts in the data [16]. This data degradation often causes models trained with high-quality laboratory data to fail to generalize when applied to real-world settings, resulting in performance degradation [17]. In particular, in medical applications, such prediction failures can have serious consequences, emphasizing the urgent need for robust BCI technologies that can maintain performance under practical constraints [18].

Despite the advancement of signal processing methods, maintaining stable BCI performance in resource-poor environments remains a challenge. Just as the human nervous system must maintain functionality despite neural noise, signal variability, and resource constraints (e.g., limited attentional capacity or sensory degradation), real-world BCIs must cope with reduced electrode density, lower sampling rates, and environmental noise [17]. Although various approaches, such as artifact removal, channel selection, and poor-quality data rejection, have been proposed, most of them still rely on relatively clean input signals. A primary factor contributing to this performance gap is the reduced sampling frequency, which is common in resource-constrained environments [19]. High sampling frequency preserves the rich temporal and spectral features of neural activity. However, low sampling frequency EEG, which is often required because of power or hardware constraints in mobile or embedded systems, fails to capture sufficient neurophysiological information [20,21]. This loss leads not only to data sparsity but also to fundamental limitations in feature representation, ultimately decreasing classification performance [22].

In this work, the researchers propose a robust framework for a filter bank common spatial pattern with time segmentation (FBCSP-TS), designed to maintain stable performance even under constraints. The proposed method segments EEG signals using optimized time intervals and applies FBCSP to each segment, enabling the cumulative extraction of spatial, spectral, and temporal features. Predictions are integrated using soft voting, which mitigates the effects of noise and segment signal degradation while effectively capturing localized and transient neural activities. Such systems embody the resilience and adaptability inherent in biological neural processing, making them more suitable for deployment in uncontrolled, user-centered environments

A primary challenge in BCI systems is achieving high accuracy with minimal resources. For validation in practical applicability and generalizability, the proposed method was applied to MI-BCI classification tasks. The proposed method demonstrates reliable classification performance even with low sampling frequencies, indicating its suitability for practical applications involving low-cost EEG devices or mobile platforms. This study contributes to laying the foundation for a robust and practical BCI system that can provide consistent performance not only in controlled laboratory settings but also in user-centered real-world settings.

Unlike conventional CSP or FBCSP methods that extract features from fixed frequency or time domains, the study’s method introduces temporal segmentation with overlapping windows, allowing the model to capture transient and distributed MI patterns more effectively. Furthermore, predictions were integrated from multiple segments through a soft voting mechanism, enhancing robustness against local signal corruption and noise. This cumulative extraction of spatial, spectral, and temporal features enables the system to maintain stable classification performance even under low sampling frequencies, a common limitation in mobile or low-cost BCI devices. By demonstrating consistent performance across two independent public datasets with varying signal quality, the proposed method offers a practical and deployable solution for EEG-based BCIs in real-world environments. Given that EEG-based MI-BCI inherently represents a biomimetic neural-interfacing technology that decodes endogenous cortical activity, improving its robustness under degraded or low-resource biological signals provides a meaningful contribution to the advancement of biomimetics. By enhancing the stability of neural feature extraction in practical environments, the proposed framework supports the development of reliable and user-centered biomimetic BCI systems.

## 2. Methods

### 2.1. Data Descriptions

The performance of the proposed methods is evaluated on two publicly available MI-BCI datasets. Details of these datasets are as follows.

#### 2.1.1. Dataset 1: BCI Competition IV Dataset 2a

**Figure 1:**
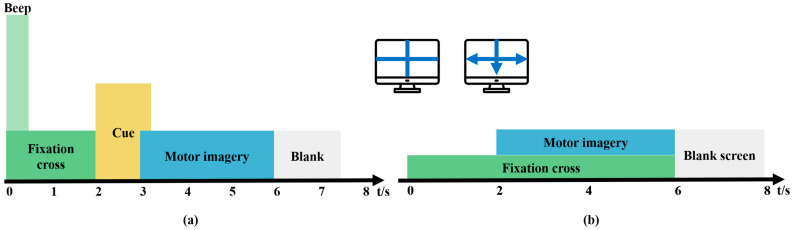
Motor imagery (MI) paradigm. (**a**) BCI Competition IV Dataset 2a, (**b**) BCI Competition IV Dataset 1.

The BCI Competition IV Dataset 2a [23] was used for training the model. This dataset contains EEG data from nine healthy subjects seated in comfortable armchairs in front of a computer screen. The cue-based BCI paradigm consists of four different MI tasks, namely, the imagination of movement of the left hand, right hand, foot, and tongue, which are the four classes. The dataset comprises 72 trials per class, making a total of 288 trials. At the start of the trial (t = 0 s), a fixation cross is shown on a black screen accompanied by a short acoustic warning tone. At t = 2 s, an arrow is shown for 1.25 s, with its direction—left, right, down, or up—corresponding to one of the four classes. The subjects were asked to perform the MI task until t = 6 s, after which a short break was given (Figure 1a). EEG signals were recorded monopolarly with the left mastoid serving as reference and the right mastoid as ground using 22 Ag/AgCl electrodes with interelectrode distances of 3.5 cm. All signals were then band-pass filtered from 0.5 to 100 Hz. The recorded EEG signals were sampled at a frequency of 250 Hz.

#### 2.1.2. Dataset 2: BCI Competition IV Dataset 1

BCI Competition IV Dataset 1 [24] was used to validate the model, and a 100 Hz downsampled version of the data was selected. Dataset 2 contains MI EEG data recorded from seven participants. These datasets were recorded from four healthy subjects (“a,” “b,” “f,” and “g”) and three computer-generated subjects (“c,” “d,” and “e”). For each subject, two classes of MI were selected from the three classes: left hand, right hand, and foot (side chosen by the subject, with both feet optional). There was a total of 100 trials for each selected class (left hand, right hand, foot), resulting in 200 trials. In the first two runs, arrows pointing left, right, or down were presented as visual cues on a computer screen. Cues were displayed for a period of 4 s, during which the subject was instructed to perform the cued MI task. These periods were interleaved with 2 s of a blank screen and 2 s of a fixation cross shown in the center of the screen. The fixation cross was superimposed on the cues for 6 s (Figure 1b). The instruments used for recording EEG signals were a BrainAmp MR Plus amplifier and an Ag/AgCl electrode cap. Signals from 59 EEG positions were measured and band-pass filtered between 0.05 and 200 Hz.

### 2.2. EEG Signal Preprocessing

The information related to MI mainly appears in the α (8–12 Hz) and β (12–32 Hz) bands of the EEG signal, whereas the ocular noise frequency range mainly exists at lower frequencies [25]. To reduce the noise of the EEG signal, a band-pass Butterworth filter with 8–32 Hz edges was applied to the data and removed artifactual interferences such as baseline drift, eye movement, and head movement.

The time window for each dataset consisted of 4 s related to the MI task and included the cue portion for the BCI IV 2a dataset. For the BCI IV 2a dataset, a data structure with the dimensions (288, 22, 1000) was created, where 288 is the total number of trials, 22 is the number of channels, and 1000 is the number of time points. For the BCI IV 1 dataset, a data structure with the dimensions (200, 59, 1000) was formed, where 200 is the total number of trials, 59 is the number of channels, and 1000 is the number of time points. These preprocessing steps provide standardized and quality data input to the classification models.

### 2.3. Data Resampling

To evaluate how signal quality degradation affects classification performance, the dataset was resampled to 25 different sampling rates ranging from 10 to 250 Hz in 10 Hz increments. Resampling was performed using the frequency-domain method implemented in the MNE-Python library (v1.9.0) [26], which applies FFT-based interpolation with built-in antialiasing. No additional filtering was required. Resampling was conducted independently along the last axis of each multidimensional signal array.

The number of samples in the resampled signal was determined by the ratio between the target and original sampling frequencies, as seen in Equation (1).

(1) $$N_{resampled}=N_{original}\left(\frac{f_{target}}{f_{original}}\right)$$

$N_{original}$ is the original number of samples, $f_{original}$ is the original sampling rate (250 Hz), and $f_{target}$ is the desired resampling rate.

This procedure enabled systematic assessment of performance stability across progressively degraded signal conditions. Statistical significance at each sampling rate was assessed using paired t-tests with Bonferroni correction to control for family-wise error rate across multiple comparisons [27]. A significance threshold of 0.05 was applied to determine the sampling frequencies at which performance degradation became statistically significant.

### 2.4. Feature Extraction

For the efficient classification of EEG signals, the characteristics of spatial, spectral, and temporal domains were applied step-by-step. These approaches aim to analyze the performance improvement effect due to the cumulative application of features at each stage.

#### 2.4.1. Common Spatial Pattern (CSP)

CSP is a widely used feature extraction algorithm that uses spatial filters to distinguish two classes for BCI [28,29]. The CSP algorithm aims to learn spatial filters that minimize the variance of a class while maximizing the variance of another. It is often helpful to band-pass filter the multichannel EEG signals [30]. For effective results, the researchers specified the frequency for band-pass filtering as 7–35 Hz. The CSP algorithm generally uses a fixed frequency range and time window through which suboptimal classification accuracy is achieved [31].

#### 2.4.2. Filter Bank Common Spatial Pattern (FBCSP)

FBCSP [32] was used to divide the EEG signal into various frequency subbands, and frequency characteristics were additionally accumulated by applying CSP to each subband. FBCSP extends the CSP approach by selecting the optimal filter band to extract EEG features by estimating the mutual information among the features in various frequency subbands. EEG signals were filtered into seven frequency bands: 4–8 Hz, 8–12 Hz, 12–16 Hz, 16–20 Hz, 20–24 Hz, 24–28 Hz, and 28–32 Hz. The optimal features were selected for classification using a mutual information-based feature selection algorithm. This method enables the detection of frequency-specific spatial patterns and improves classification performance by exploiting MI-related oscillations in the μ and β bands.

### 2.5. Proposed Method

#### Filter Bank Common Spatial Pattern with Time Segmentation (FBCSP-TS)

**Figure 2:**
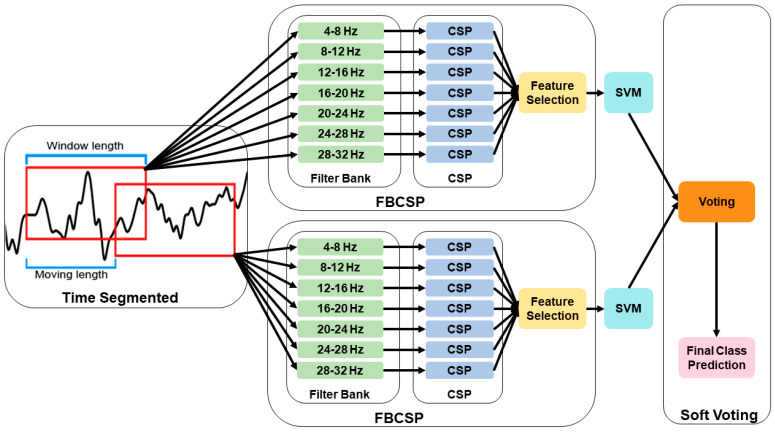
Flowchart of the filter bank common spatial pattern with time segmentation (FBCSP-TS) methodology.

FBCSP-TS was applied to search for the time segmentation with optimized performance, and through this, temporal features were additionally accumulated (Figure 2). Data segmentation was applied to improve the robustness of feature extraction at low sampling frequencies. The researchers identified transient temporal features by applying the FBCSP method across multiple overlapping time segments. Specifically, the researchers tested a comprehensive combination of window lengths (0.5–4.0 s) and moving steps (0.5–4.0 s), yielding 64 distinct temporal configurations. The window length represents the temporal interval for feature extraction, whereas the moving length defines the temporal displacement between consecutive analysis windows. Classification performance was evaluated across all combinations to identify the optimal temporal segments for MI and visualized using a heatmap. This parametric design allowed the model to capture optimal temporal segments for classification. Notably, the use of overlapping temporal segments (i.e., shorter moving steps than window lengths) has been shown to enhance signal-to-noise ratio (SNR) and subsequently improve classification performance [33,34]. A soft voting method was applied to address the dimensional mismatch between the segmented data and the original data. The final class was determined by averaging the predicted probabilities of each time segment, thereby producing consistent classification results that reflected the information of all segments.

### 2.6. Classification and Evaluation Metrics

EEG signal classification was performed using a support vector machine (SVM) [35] with a radial basis function (RBF) kernel. SVM is a widely used supervised learning algorithm for neurophysiological data because it determines an optimal decision boundary while maintaining robustness against overfitting [36]. It is effective for high-dimensional EEG feature spaces [37] and performs reliably even with relatively small numbers of training trials, which is common in BCI datasets [38,39]. The target task in this study involved four motor imagery (MI) classes (left hand, right hand, foot, and tongue). SVM was selected to provide a stable and comparable baseline, as the combination of CSP-based methods and SVM classifiers is widely regarded as a benchmark approach in MI-BCI research [40,41].

The performance of the proposed method was evaluated using accuracy and Cohen’s kappa coefficient. Accuracy refers to the correctly classified proportion of all samples, and Cohen’s kappa coefficient can complementarily measure model reliability by correcting the possibility of simple accidental agreement. Where P(e) is the hypothetical probability of chance agreement, the observed data is used to calculate the probabilities of each observer randomly saying each category, as seen in Equations (2) and (3).

(2) $$Accuracy=\frac{TP+TN}{TP+TN+FP+FN}$$

(3) $$Kappa=\frac{accuracy-p_{e}}{1-p_{e}}$$

Fivefold cross-validation was applied to ensure the fairness and accuracy of the evaluation. The generalization performance of the model was confirmed by repeatedly performing learning and verification at each fold, and the final result was calculated as the average performance at all folds.

### 2.7. External Validation on an Independent Dataset

To evaluate the generalizability of the proposed method, the researchers performed external validation using the BCI Competition IV Dataset 1. This dataset consists of EEG signals collected at a lower sampling frequency (100 Hz) than the training dataset (250 Hz). The researchers applied the same preprocessing, feature extraction, and classification pipelines to this independent dataset to evaluate the robustness and applicability of the proposed approach. Notably, Dataset 1 contains EEG recordings of both real subjects (“a,” “b,” “f,” and “g”) and artificially generated subjects (“c,” “d,” and “e”). The classification performance was compared across three methods: CSP, FBCSP, and the proposed FBCSP-TS. The performance was quantified using both accuracy and Cohen’s kappa coefficient.

## 3. Results

**Figure 3:**
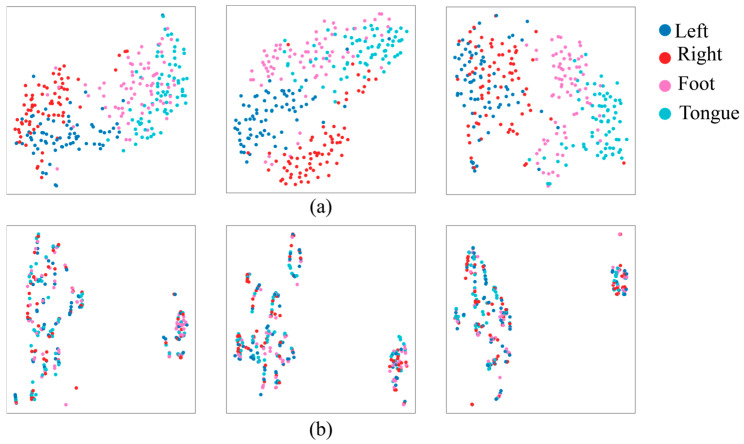
Differences between electroencephalography (EEG) feature distribution at (**a**) 250 Hz and (**b**) 10 Hz sampling frequencies.

Figure 3 shows the data of the original frequency of 250 Hz and the lowest frequency of 10 Hz, which were represented in a 2D space using the t-distributed stochastic neighbor embedding (t-SNE) [42] technique to analyze the effect of the sampling frequency of EEG signals on the classification performance. Blue, red, pink, and cyan dots represent the left hand, right hand, foot, and tongue, respectively. At 250 Hz, it can be seen that the four classes (left hand, right hand, foot, and tongue) are separated. On the other hand, at 10 Hz, it can be seen that all classes are distributed in a disorderly manner, and the possibility of discrimination between classes is lowered. The results show that class classification is better with higher sampling frequencies, and lower sampling frequencies adversely affect class classification.

**Figure 4:**
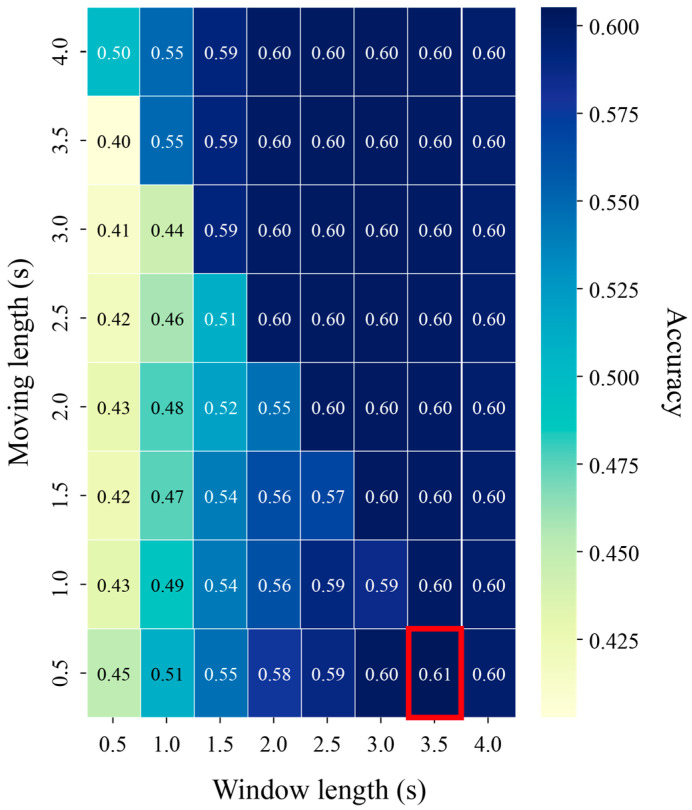
Heatmap showing classification accuracy across different time segmentations using BCI Competition IV Dataset 2a.

Figure 4 shows the heatmap visualization of the classification performance according to the time division parameters to distinguish MI using BCI Competition IV Dataset 2a. The matrix shows the maximum accumulation value achieved by the machine learning model, the column is the window length, and the row is the moving length. In the phase triangular matrix section where the sum of the window length and the travel length exceeds 4 s, the signal cannot be further divided, so only the same data of the first window length is used to show the same performance. The red rectangle shows the optimal time interval for each classification task. A maximum performance of 0.61 was achieved when using the SVM classifier in a window length of 3.5 s and a moving length of 0.5 s.

**Figure 5:**
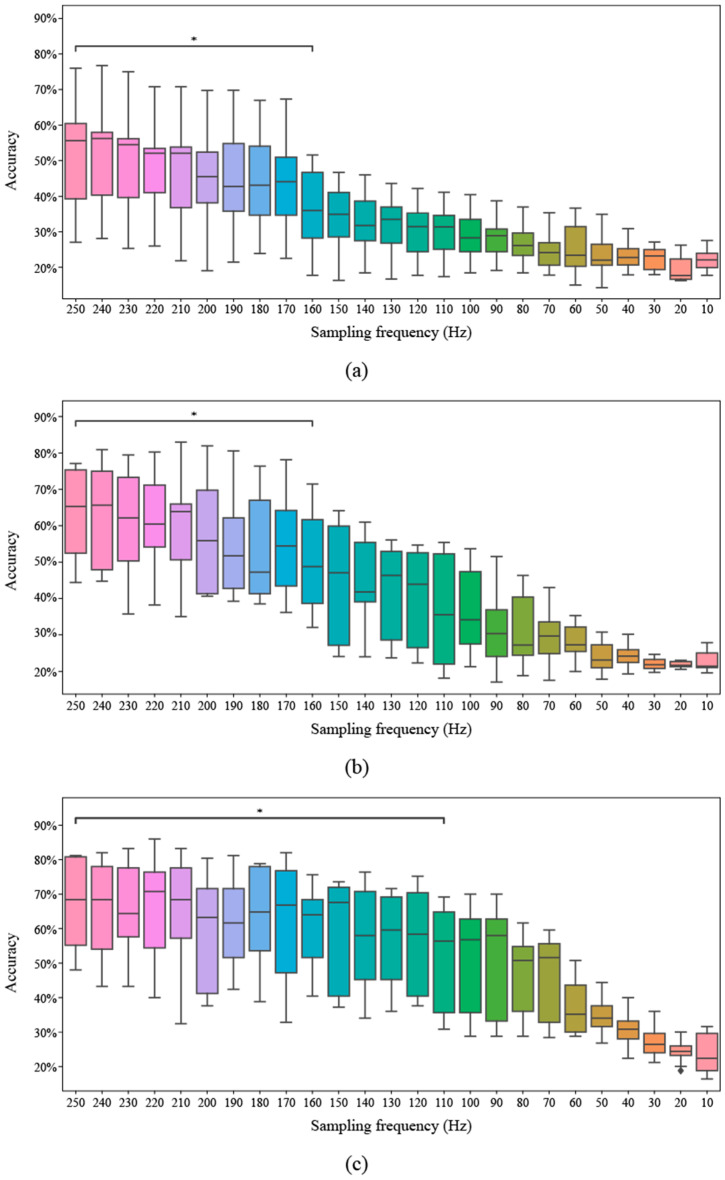
Classification accuracy across different sampling frequencies for three methods. The asterisk (*) indicates a *p*-value lower than 0.05. (**a**) Common spatial pattern (CSP), (**b**) Filter bank common spatial pattern (FBCSP), (**c**) Filter bank common spatial pattern with time segmentation (FBCSP-TS).

Figure 5 below shows the classification performance according to the sampling frequency. Figure 5a shows CSP, Figure 5b shows the FBCSP combining the CSP with the frequency features, and Figure 5c shows the FBCSP-TS performance using the optimal time 3.5 s window length and 0.5 s moving length for the FBCSP. The median values are CSP: 0.58, FBCSP: 0.65, and FBCSP-TS: 0.68, and the proposed method shows higher performance than CSP and FBCSP. In addition, CSP and FBCSP maintained performance up to 160 Hz, while the FBCSP-TS technique showed stable performance up to 110 Hz and maintained high accuracy even at low sampling frequencies. It was shown that the more the frequency and time were added step-by-step to the general CSP, the longer the performance maintenance period was.

**Figure 6:**
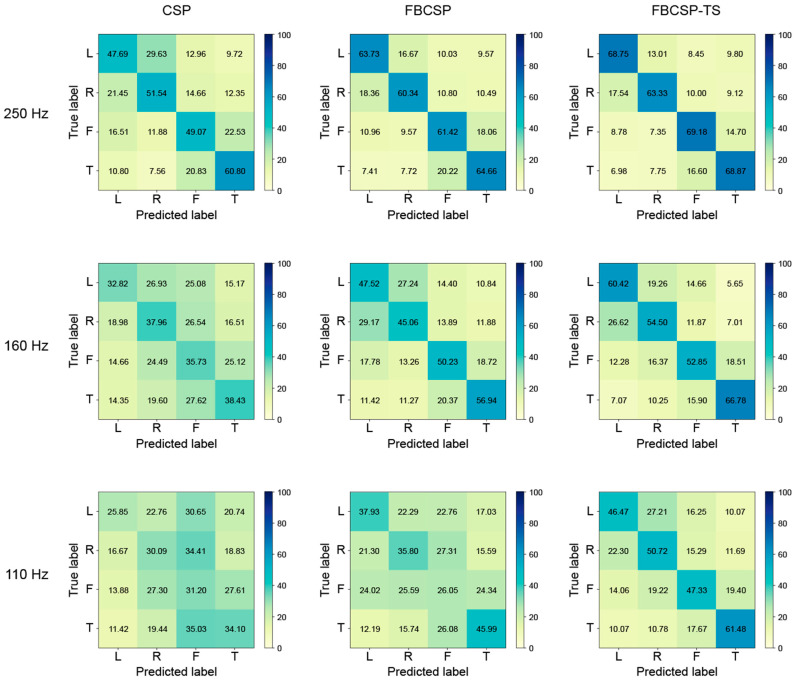
Confusion matrices for common spatial pattern (CSP), filter bank common spatial pattern (FBCSP), and filter bank common spatial pattern with time segmentation (FBCSP-TS) in three sampling frequencies (250 Hz, 160 Hz, 110 Hz).

In addition, the researchers compared the classification performance of three methods (CSP, FBCSP, and FBCSP-TS) through a confusion matrix, and the results are presented in Figure 6. Each method was performed under three sampling frequency conditions of 250 Hz, 160 Hz, and 110 Hz, and for each sampling frequency, an interval was selected where statistical significance was confirmed based on 250 Hz in the CSP and FBCSP-TS methods. At the sampling frequency of 250 Hz initially recorded in the EEG signal, all three methods showed relatively good diagonal dominance in the confusion matrix. In particular, all methods recorded an accuracy of over 60% for the tongue class. Overall, confusion occurred relatively frequently between the left and right classes and the foot and tongue classes. Still, the FBCSP and FBCSP-TS methods, which additionally reflected frequency and time information, showed a pattern in which the misclassification rate decreased, and the proportion of the main diagonal component increased. Statistical differences occurred in the 160 Hz section in the CSP method, and all classes showed about 30% accuracy. On the other hand, FBCSP and FBCSP-TS methods maintained their original performance relatively well, and in particular, FBCSP-TS only showed about a 2% accuracy difference in the tongue class. At the lowest sampling frequency of 110 Hz, all methods showed a decrease in classification performance, but the degree of the decrease differed. CSP showed about 30% accuracy in all classes, CSP showed about 25% accuracy in all classes, and FBCSP showed about 25% accuracy in the foot class, indicating that the model did not function properly. FBCSP-TS maintained relatively robust performance even in the section where statistically significant differences occurred. In particular, the method showed a similar level of performance to CSP at 250 Hz and FBCSP at 160 Hz.

The researchers compared its performance using the BCI Competition IV Dataset 1, which was recorded at a low sampling frequency of 100 Hz, to validate the proposed method (Table 1). The proposed method achieved the highest classification accuracy in five out of seven subjects, excluding subjects “c” and “d.” In addition, the average classification accuracy was 0.809, which is higher than that of FBCSP (0.779) and CSP (0.511). Based on the CSP, adding frequency features improved the performance by 0.268, and integrating both frequency and time features improved the performance by 0.298. Moreover, the proposed method yielded the highest mean kappa value (0.619), indicating stronger reliability in classification outcomes. In contrast, CSP (0.032) and FBCSP (0.560) showed lower agreement, with some subjects (“c,” “e,” and “g”) even exhibiting negative kappa under CSP. These results demonstrate the effectiveness of the proposed method in terms of performance improvement at a low sampling frequency.

## 4. Discussion

The primary objective of this study was to propose a method that maintains classification performance in an EEG-based BCI system under low sampling frequency conditions by progressively incorporating spatial, spectral, and temporal features. This approach addresses a critical limitation in practical BCI systems, where hardware or processing constraints often lead to signal degradation, thereby compromising model reliability.

Through t-SNE visualization, the impact of sampling frequency on EEG classification performance was clearly demonstrated. At high resolution (250 Hz), the separation among the four MI classes was distinct, whereas at low resolution (10 Hz), class boundaries became blurred, making accurate classification more challenging. This finding highlights the importance of sampling rate in preserving the spatiotemporal structure inherent in EEG signals.

The researchers further investigated the temporal segments that best captured MI characteristics. EEG signals were segmented using various combinations of window and moving lengths, and model performance was compared accordingly. The optimal performance was observed at a window length of 3.5 s with a moving length of 0.5 s. This finding aligns with neurophysiological evidence indicating that changes in μ (8–13 Hz) and β (13–30 Hz) rhythms occur in localized and transient patterns during MI tasks [43]. These changes are most pronounced within 1–3 s following cue presentation, emphasizing the need for adequate temporal resolution. Utilizing overlapping segments not only enhances the stability in capturing such transient signals but also improves the SNR [33].

Moreover, the researchers evaluated the classification stability across varying sampling frequencies using three methods: CSP, FBCSP, and FBCSP-TS. While CSP and FBCSP maintained performance down to 160 Hz, the proposed method retained high accuracy even at 110 Hz. These differences stem from each method’s capacity to preserve signal information under degradation. CSP, limited to fixed frequency and temporal domains, exhibits rapid performance deterioration as noise becomes more dominant. FBCSP partly compensates for this by incorporating multiple frequency bands, yet still struggles under low-resolution signals. In contrast, the FBCSP-TS method, by decomposing signals into overlapping temporal segments, minimizes information loss and exhibits robust performance. The key contribution of FBCSP-TS is not that it improves performance at low frequencies compared to high frequencies, but rather that it maintains more stable and functional performance across a wider range of degraded conditions compared to conventional methods. These differences stem from each method’s capacity to preserve signal information under degradation. Extracting features independently from multiple segments introduces diversity and resilience in the information, compensating for signal degradation more effectively than frequency-based methods. This segmented structure enables the model to focus on informative intervals within the signal, thereby sustaining classification performance. Furthermore, prediction outputs from each segment are aggregated via a soft voting scheme, mitigating the influence of noise or signal corruption in any single segment and enhancing model robustness.

Confusion matrices provided detailed insights into class-specific performance. Analysis of the confusion matrix can help evaluate classification consistency and predictive stability. In MI-BCI systems, reliable classification of each MI class is critical, as it directly influences the system’s practical usability. The proposed method not only improved overall accuracy but also equalized class-specific performance. At 250 Hz, misclassifications were primarily observed between the left and right classes and the foot and tongue classes. However, the method significantly reduced misclassification rates compared to CSP and FBCSP. Even at 110 Hz, it maintained a relatively balanced performance across all classes, indicating enhanced consistency and meaningful information extraction from low-quality signals.

The researchers also validated the generalizability of the proposed FBCSP-TS using the independent BCI Competition IV Dataset 1. This dataset differs from the training data in experimental settings, channel configurations, and task structures. Hence, the evaluation serves to assess the model’s adaptability to new users, devices, and protocols. The method achieved a mean accuracy of 0.809 and a Cohen’s kappa coefficient of 0.619, outperforming CSP (0.211, 0.032) and FBCSP (0.779, 0.560). Notably, the best performance was observed in five non-synthetic subjects, indicating that the model is not overfitted to specific datasets but demonstrates practical applicability to real-world EEG recordings. This finding suggests that the method could serve as a reliable foundation for developing deployable BCI systems.

Nevertheless, several practical challenges remain before clinical application. First, although the proposed method was validated on an independent dataset, all analyses were performed offline using resting-state EEG, and future work should examine real-time applicability under dynamic, mobile, or rehabilitation environments where movement and noise are present. Furthermore, real-world EEG degradation is not caused solely by hardware limitations; user-state variability such as attentional fluctuations, fatigue, stress, and emotional changes can also disrupt MI patterns. The proposed method does not fully eliminate these intrinsic nonstationarities, although temporal segmentation may mitigate brief disruptions by distributing decisions across multiple windows. Comprehensive handling of user-related variability will require adaptive or multimodal approaches, which we identify as an important direction for future research. Second, the resampling procedure relied on FFT-based interpolation, which inherently includes antialiasing. Real-world systems would experience additional aliasing, and our findings represent a conservative estimate of performance loss. Future work should validate the proposed method using EEG recorded natively at low sampling frequencies or through hardware-level emulation to fully incorporate the effects of real-world aliasing and amplifier characteristics. Third, this study did not include comparisons with classifier-level optimization approaches. These techniques [44] target improvements at the decision-boundary level, whereas our approach focuses on feature-level robustness through temporal segmentation. Importantly, the temporal segmentation framework is classifier-agnostic and could, in principle, be integrated with state-of-the-art methods such as Riemannian-geometry-based classifiers or lightweight deep-learning architectures, which have shown strong robustness under noisy or nonstationary EEG conditions. Exploring how temporal segmentation interacts with these modern approaches may provide a more comprehensive understanding of how combined feature- and classifier-level strategies improve MI-BCI performance under degraded or real-world conditions.

## 5. Conclusions

In this study, the researchers proposed a novel framework for BCI systems that maintains high performance even with low-quality EEG signals. By integrating spatial, spectral, and temporally segmented features, the proposed method effectively compensates for information loss caused by signal quality degradation, thereby extending the performance retention range. Through evaluation across various sampling frequencies, the approach not only achieved higher accuracy than traditional CSP and FBCSP methods but also demonstrated a longer performance maintenance range. Furthermore, confusion matrix analysis and generalization to external datasets confirmed the practical applicability and reproducibility of the proposed BCI system. By minimizing dependency on high-quality data while maintaining robust classification performance, this methodology offers a significant contribution to the BCI system. In particular, enabling the use of low-quality EEG equipment without compromising accuracy substantially improves the practicality and accessibility of BCI in real-world settings. This study highlights the potential for developing robust and deployable BCI solutions applicable across a variety of real-world settings, including mobile, clinical, and home-based applications.

## Figures and Tables

**Table 1:** Comparison of classification accuracy and kappa for common spatial pattern (CSP), filter bank common spatial pattern (FBCSP), and filter bank common spatial pattern with time segmentation (FBCSP-TS) using Dataset 2 (BCI Competition IV Dataset 1).

Subject	CSP		FBCSP		FBCSP-TS	
	Accuracy	Kappa	Accuracy	Kappa	Accuracy	Kappa
a	0.542	0.095	0.785	0.570	0.840	0.680
b	0.527	0.061	0.534	0.070	0.644	0.290
c	0.487	−0.019	0.715	0.430	0.705	0.410
d	0.502	0.001	0.860	0.720	0.805	0.610
e	0.472	−0.042	0.865	0.730	0.909	0.820
f	0.577	0.161	0.865	0.730	0.875	0.750
g	0.472	−0.030	0.835	0.670	0.890	0.779
mean	0.511	0.032	0.779	0.560	0.809	0.619

## Data Availability

The data presented in this study are publicly available (https://www.bbci.de/competition/iv/), accessed on 15 June 2025.

## Abbreviations

The following abbreviations are used in this manuscript:

BCI	Brain–Computer Interface
CSP	Common Spatial Pattern
EEG	Electroencephalography
FBCSP	Filter Bank Common Spatial Pattern
FBCSP-TS	Filter Bank Common Spatial Pattern with Time Segmentation
FFT	Fast Fourier Transform
fMRI	Functional Magnetic Resonance Imaging
MI	Motor Imagery
MRI	Magnetic Resonance Imaging
SNR	Signal-to-Noise Ratio
SSVEP	Steady-State Visual Evoked Potential
SVM	Support Vector Machine
t-SNE	t-Distributed Stochastic Neighbor Embedding
